# Bone Morphogenetic Protein 4 (BMP4) Enhances the Differentiation of Human Induced Pluripotent Stem Cells into Limbal Progenitor Cells

**DOI:** 10.3390/cimb43030147

**Published:** 2021-11-29

**Authors:** Hyun Soo Lee, Jeewon Mok, Choun-Ki Joo

**Affiliations:** 1 Department of Ophthalmology, Eunpyeong St. Mary’s Hospital, College of Medicine, The Catholic University of Korea, Seoul 03312, Korea; 2 Department of Ophthalmology, Seoul St. Mary Hospital, College of Medicine, The Catholic University of Korea, Seoul 03312, Korea; mjwcatholic@gmail.com (J.M.); ckjoocatholic@gmail.com (C.-K.J.)

**Keywords:** bone morphogenetic protein 4 (BMP4), cornea, induced pluripotent stem (iPS) cells, limbal progenitor cells, limbal stem cell deficiency (LSCD)

## Abstract

Corneal epithelium maintains visual acuity and is regenerated by the proliferation and differentiation of limbal progenitor cells. Transplantation of human limbal progenitor cells could restore the integrity and functionality of the corneal surface in patients with limbal stem cell deficiency. However, multiple protocols are employed to differentiate human induced pluripotent stem (iPS) cells into corneal epithelium or limbal progenitor cells. The aim of this study was to optimize a protocol that uses bone morphogenetic protein 4 (BMP4) and limbal cell-specific medium. Human dermal fibroblast-derived iPS cells were differentiated into limbal progenitor cells using limbal cell-specific (PI) medium and varying doses (1, 10, and 50 ng/mL) and durations (1, 3, and 10 days) of BMP4 treatment. Differentiated human iPS cells were analyzed by real-time polymerase chain reaction (RT-PCR), Western blotting, and immunocytochemical studies at 2 or 4 weeks after BMP4 treatment. Culturing human dermal fibroblast-derived iPS cells in limbal cell-specific medium and BMP4 gave rise to limbal progenitor and corneal epithelial-like cells. The optimal protocol of 10 ng/mL and three days of BMP4 treatment elicited significantly higher limbal progenitor marker (ABCG2, ∆Np63α) expression and less corneal epithelial cell marker (CK3, CK12) expression than the other combinations of BMP4 dose and duration. In conclusion, this study identified a successful reprogramming strategy to induce limbal progenitor cells from human iPS cells using limbal cell-specific medium and BMP4. Additionally, our experiments indicate that the optimal BMP4 dose and duration favor limbal progenitor cell differentiation over corneal epithelial cells and maintain the phenotype of limbal stem cells. These findings contribute to the development of therapies for limbal stem cell deficiency disorders.

## 1. Introduction

The cornea is a transparent and multilayered structure of the eye that is essential for transmitting and focusing light to the retina. The corneal epithelium consists of non-keratinized, stratified squamous epithelial cells. These cells are continuously regenerated by limbal progenitor cells that reside in the basal layer of the limbus [1,2]. Chemical and thermal burns, contact lenses, radiotherapy, microbial infections, sulfur mustard gas exposure, congenital aniridia, and chronic inflammatory diseases (e.g., Stevens-Johnson syndrome or ocular cicatricial pemphigoid) cause severe limbal stem cell deficiency (LSCD). LSCD symptoms progress from corneal epithelial defects, opacity, neovascularization, and fibrovascular pannus to visual impairment [3,4,5]. Current surgical therapies for LSCD include limbal tissue transplantation, amniotic membrane transplantation, and corneal epitheliopathy. However, sources of limbal tissue are limited. Cell-based alternatives for limbal tissue transplantation, such as autologous limbal epithelial or oral mucosal epithelial cells that have undergone ex vivo culture, have been reported to relieve LSCD [3,5]. However, these surgical procedures are complex and time-consuming, and the visual prognosis of patients treated to date has been unsatisfactory [1,3,4].

Recent studies have shown that induced pluripotent stem (iPS) cells can be generated from somatic cells such as dermal fibroblasts and are capable of pluripotent differentiation and indefinite expansion in vitro [5]. Therefore, iPS cells are a potentially unlimited source to replace damaged target cells or be differentiated into progenitors of target cells for regenerative medicine [6,7]. BMP4 promotes human iPS cell differentiation into surface ectodermal cells, such as dental epithelium and stratified epithelial cells [8].

Bone morphogenetic protein 4 (BMP4) plays a vital role in eye development [9,10]. BMP4 is expressed in corneal epithelium and participates in corneal epithelial cell proliferation [10,11,12]. BMP4 is also required to differentiate iPS cells into corneal epithelial cells [11,12,13]. However, the most effective dose and duration of BMP4 to differentiate human iPS cells into limbal progenitor cells remain unknown.

This study was designed to optimize an in vitro culture model for differentiating human iPS cells into limbal progenitor cells, using limbal cell-specific culture media and BMP4 treatment.

## 2. Materials and Methods

### 2.1. Human iPS Cell Culture

All experiments were carried out in accordance with our institutional research guidelines. Human iPS cells were cultured according to a previously described method and previously well confirmed by the provider [8]. Briefly, the adult human dermal fibroblast-derived human iPS cell line (201B7) and PA6 cell line (as feeder cells, RCB1127) were purchased from the RIKEN Bioresource Center (Ibaraki, Japan). Human iPS cells were grown on a radiation-treated mouse embryonic fibroblast (MEF) feeder layer in standard iPS culture medium, containing DMEM/F12 (Invitrogen, Carlsbad, CA, USA) with 20% knockout serum replacement (Invitrogen), 0.1 mM 2-mercaptoethanol (Invitrogen), 100 U/mL penicillin/streptomycin (Invitrogen), and 1% non-essential amino acids (Invitrogen). Cells were passaged every week, and the medium was changed every two days [14].

### 2.2. Differentiation of Human iPS Cells into Limbal Progenitor Cells

Human iPS cells were harvested and then seeded on fibronectin and laminin-coated 6-well plate (BD Biosciences, San Diego, CA, USA) at a density of 1× 10^4^ cells per well with limbal-specific medium (referred as PI medium) according to a previously described method [15,16]. In brief, Iscove′s Modified Dulbecco’s Medium (IMDM) (Sigma-Aldrich, St. Louis, MO, USA) with 10% fetal bovine serum (FBS; Invitrogen) was mixed with complete Panserin 801 medium in a 1:1 ratio. Complete Panserin 801 medium contains bovine pituitary extract, epidermal growth factor, hydrocortisone, insulin, phosphoethanolamine, and ethanolamine, which are provided as supplements by the manufacturer (P04-710801K, Pan Biotech, Aidenbach, Germany). Cells were daily treated with BMP4 (R&D System, Minneapolis, MN, USA) at 1, 10, or 50 ng/mL for the first 1, 3, or 7 days of culture in PI medium. Then, PI medium was changed every 2 to 3 days for one month.

### 2.3. RNA Isolation and Real-Time Polymerase Chain Reaction (RT-PCR)

Total RNA was isolated from differentiated human iPS cells aggregates at day 30 of each differentiation protocol using Trizol (Invitrogen) and RNeasy micro-kits (Qiagen, Valencia, CA, USA). Complementary DNA was then synthesized using SuperScript IIITM reverse transcriptase (Invitrogen, CA, USA), and quantitative RT-PCR was performed using Taqman Universal PCR Mastermix (applied biosystems, Foster, MA, USA) and FAM-dye-labeled customized primers (Macrogen, Seoul, Korea). The following primer sequences were used: Cytokeratin (CK) 3 (sense, 5′-CTTCAACACCCGCTTTGTCA-3′; antisense, 5′-GGAGACAGACACCTTTCCCA-3′), CK12 (sense, 5′-TTCTGCTGCTTCCATGTTTG-3′; antisense, 5′-TCATTGCCCGAGAGAATACC-3′), ABCG2 (sense, 5′-ACCATTGCATCTTGGCTGTC-3′; antisense, 5′-CGATGCCCTGCTTTACCAAA-3′), ∆Np63α (sense, 5′-GCATTGTCAGTTTCTTAGCGAG-3′; antisense, 5′-CCATGGAGTAATGCTCAATCTG-3′), and glyceraldehyde 3-phosphate dehydrogenase (GAPDH) (sense, 5′-GCCAAGGTCATCCATGACAAC-3′; antisense, 5′-GTCCACCACCCTGTTGCTGTA-3′). One µL of cDNA was loaded into each well, and the GAPDH gene was used as an endogenous reference. The results were analyzed by the comparative threshold cycle (CT) method, and the relative expression level of each gene was expressed as the fold change relative to control cells (iPS cells grown only in PI-medium without BMP4).

### 2.4. Western Blot Assays

Differentiated human iPS cells were harvested after one month of culture in PI medium by scraping them into RIPA buffer (Thermo Fisher Scientific, San Jose, MA, USA). Cell lysates were incubated for 2 h at 4 °C and then centrifuged. Protein concentrations in the supernatants were determined using a BCA assay Kit (Thermostat, Hercules, CA, USA), and equal amounts of protein were separated by 10% SDS-PAGE (Whatman, Inc., Clifton, NJ, USA). The proteins were then transferred onto a PVDF membrane (Millipore Corporation, Billerica, MA, USA) and incubated with primary antibodies at 4 °C overnight. Primary antibodies were detected by adding secondary antibodies conjugated to horseradish peroxidase (HRP) (Santa Cruz Biotechnology, Dallas, TX, USA) and incubating for 1 h. The membrane was then washed five times, and chemiluminescence was detected using the Immobilon Western substrate (Millipore Corporation, Billerica, MA, USA) and a ChemiDoc MP Imaging System (BioRad Laboratories Inc., Hercules, CA, USA). Antibodies and their dilutions are shown in Table 1.

### 2.5. Immunohistochemical Staining

The following primary antibodies were used for immunohistochemical staining: goat anti-human CK3 (1:200; BD Pharmingen, San Diego, CA, USA) and mouse anti-human ABCG2 (1:100; Millipore, Burlington, MA, USA). DyLight 488-conjugated anti-goat IgG antibody (Abcam, Boston, MA, USA) and DyLight 649-conjugated anti-mouse IgG antibody (Abcam) were used as secondary antibodies. Differentiated human iPS cells were fixed with 4% paraformaldehyde for 20 min and then incubated with 5% donkey serum and 0.3% Triton X for 1 h to block non-specific binding. Fixed cells were immunostained with primary antibodies or isotype antibodies at 4 °C overnight and then exposed to secondary antibodies. Samples were mounted using Vector Shield mounting medium with 4,6 diamidino-2-phenylindole (DAPI, Vector Laboratories, Burlingame, CA, USA) and observed under an epifluorescent microscope (Axiovert 200, Carl Zeiss, Jena, Germany) at 100× magnification. The percentage of positive cells for each marker was quantified relative to the number of DAPI-stained cells from randomly selected areas in each plate. The analysis was performed in a blinded fashion [17].

### 2.6. Statistical Analysis

Experiments were performed three times, and results were expressed as means ± standard deviation (SD). Statistical significance was analyzed by one-way analysis of variance (ANOVA) followed by Tukey’s post-hoc test or Student’s *t*-test using Prism software (version 5.0; GraphPad, San Diego, CA, USA). *p* < 0.05 was regarded as significant.

## 3. Results

### 3.1. Differentiation of Human iPSC into Corneal Limbal Progenitor Cells Using Varying Doses of BMP4

**Figure 1:**
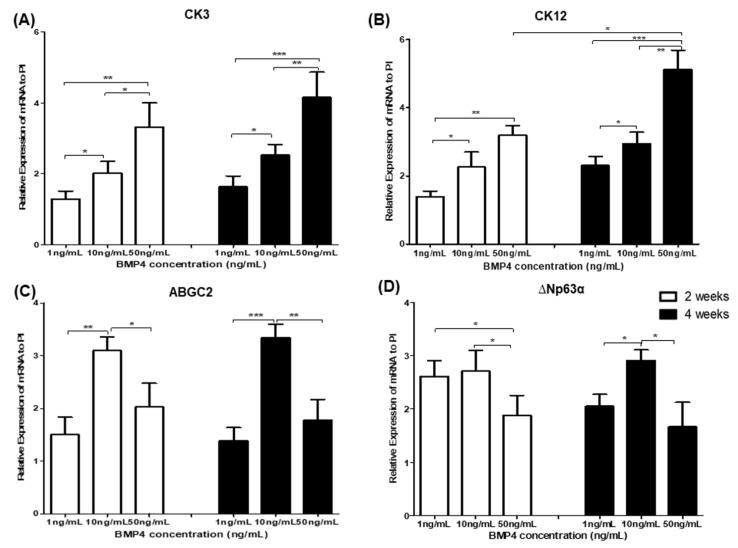
Analysis of gene expression for corneal epithelium markers (**A**,**B**) and limbal progenitor-related markers (**C**,**D**) in human iPS cells grown in a limbal cell culture system with varying doses of BMP4. Human iPS cells were harvested and seeded on fibronectin and laminin-coated dishes with a limbal-specific medium (PI). BMP4 (1, 10, and 50 ng/mL) was present for the first three days of culture. Data represent 3 or 4 independent experiments and are presented as means ± standard deviation (* *p* < 0.05; ** *p* < 0.001; *** *p* < 0.001).

We monitored the differentiation of human iPS cells into corneal limbal progenitor cells using varying doses and durations of BMP4 stimulation to establish the optimal BMP4 regimen. The differentiation of human iPS cells into corneal limbal progenitors was evaluated at 2- and 4-weeks incubation in PI medium after receiving different doses of BMP4 (1, 10, and 50 ng/mL) for 3 days (Figure 1). PI medium was previously demonstrated to support in vitro corneal limbal cell culture [9]. BMP4 induced human iPS cells to differentiate into corneal epithelial-like cells (CK3 or CK12) in a dose-dependent manner: 10 and 50 ng/mL BMP4 yielded significantly more corneal epithelial-like cells than 1 ng/mL at 2 and 4 weeks (*p* < 0.05, 1 ng/mL vs. 10 ng/mL BMP4 at 4 weeks, and *p* < 0.001, 1 ng/mL vs. 50 ng/mL BMP4 at 4 weeks: Figure 1A,B, respectively). However, BMP4 differentiated human iPS cells into corneal limbal progenitor cells (ABCG2 or ∆Np63α) in a dose-nondependent manner. Using 10 ng/mL BMP4 increased the expression of ABCG2 significantly more than 1 or 50 ng/mL BMP4 (*p* < 0.0001, 10 ng/mL vs. 1 ng/mL BMP4 at 4 weeks, and *p* < 0.01, 10 ng/mL vs. 50 ng/mL BMP4 at 4 weeks, respectively; Figure 1C). In addition, 10 ng/mL BMP4 stimulation induced more ∆Np63α expression than 1 or 50 ng/mL BMP4 (*p* < 0.05, 10 ng/mL vs. 1 ng/mL BMP4 at 4 weeks, and *p* < 0.05, 10 ng/mL vs. 50 ng/mL BMP4 at 4 weeks, respectively; Figure 1D). Moreover, 50 ng/mL BMP4 induced significantly more CK12 at 4 weeks than at 2 weeks (*p* < 0.05, 2 weeks vs. 4 weeks; Figure 1B).

### 3.2. Limbal Progenitors Differentiation from Human iPS Cells in Different Durations of BMP4 Treatment

**Figure 2:**
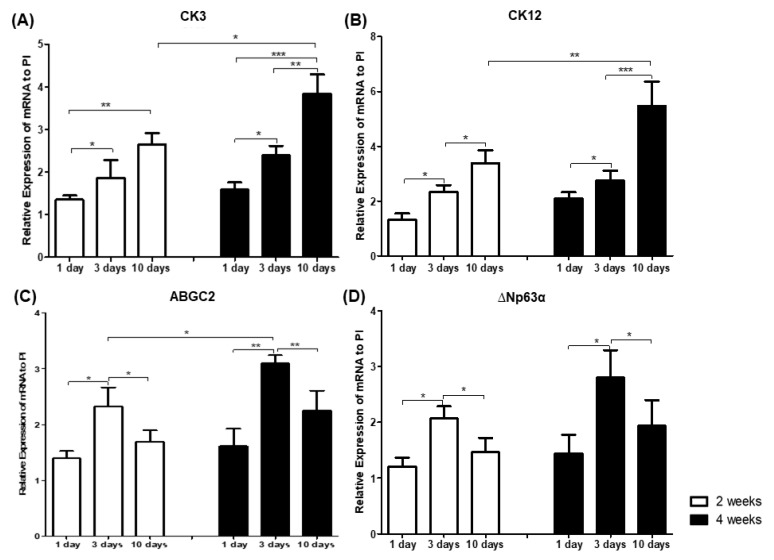
Analysis of gene expression analysis for corneal epithelium markers (**A**,**B**) and limbal progenitor-related markers (**C**,**D**) in human iPS cells grown in a limbal cell culture system with varying durations of BMP4 administration. Human iPS cells were cultured in a limbal-specific medium (PI) with 10 ng/mL BMP4 during the first 1, 3, or 10 days of culture. Data represent 3 or 4 independent experiments and are presented as means ± standard deviation (* *p* < 0.05; ** *p* < 0.001; *** *p* < 0.001).

The differentiation of human iPS cells into corneal limbal progenitors after 1, 3, or 10 days of 10 ng/mL BMP4 treatment was evaluated at 2 and 4 weeks in PI medium (Figure 2). BMP4 increased the differentiation of human iPS cells into corneal epithelial-like cells (CK3 and CK12) in a time-dependent manner (*p* < 0.05, 1 day vs. 3 days at 4 weeks, and *p* < 0.001, 3 days vs. 10 days at 4 weeks: Figure 2A,B, respectively). However, BMP4 did not induce the differentiation of human iPS cells into corneal limbal progenitor cells (ABCG2 or ∆Np63α) in a time-dependent manner. Three days of BMP4 stimulation produced significantly more ABCG2 and ∆Np63α expression than 1 or 10 days (*p* < 0.001, 3 days vs. 1 or 10 days at 4 weeks, and *p* < 0.05, 3 days vs. 1 or 10 days at 4 weeks; Figure 2C,D, respectively). Furthermore, 10 days of BMP4 administration induced significantly more CK3 and CK12 at 4 weeks than at 2 weeks in PI medium (*p* < 0.05, 2 weeks vs. 4 weeks, and *p* < 0.001, 2 weeks vs. 4 weeks; Figure 2A,B, respectively). In addition, 3 days of 10 ng/mL BMP4 treatment produced significantly more ABCG2 at 4 weeks in PI medium than at 2 weeks (*p* < 0.05, 2 weeks vs. 4 weeks; Figure 2C).

### 3.3. Morphology of Differentiated Human iPS Cells Treated with the Optimal Dose and Duration of BMP4

**Figure 3:**
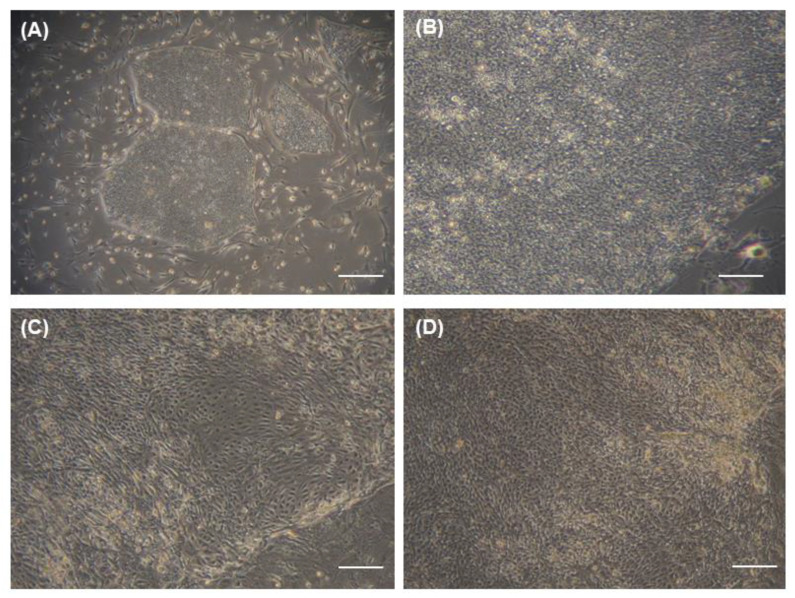
Morphologic changes of cultured human iPS cells. Bright-field microscopy images of undifferentiated human iPS cell cultures at (**A**) low-magnification (10×); scale bar = 500 μm) and (**B**) high-magnification (100×). Bright-field microscopy images of human iPS cells differentiated in (**C**) limbal-specific (PI) medium only (100×) and (**D**) PI medium with the optimal BMP4 treatment (100×). Scale bar = 50 μm (**B**–**D**).

Human iPS cells (Figure 3A,B) exhibited the typical morphology of iPS cells: round colonies of flat, dense cells with scant cytoplasm and prominent nucleoli [18]. Partially differentiated human iPS cells cultured in PI medium alone were heterogeneous populations of large, ovoid, fusiform cells (Figure 3C). However, human iPS cells cultured with PI medium with 10 ng/mL BMP4 for the first 3 days exhibited a limbal cell-like morphology: small, cuboidal, compact, cobblestone-like, regularly arranged cells that were more homogeneous than those grown in PI medium alone at 4 weeks (Figure 3D) [19].

### 3.4. Effects of BMP4 Treatment on Limbal Cell Marker Expression

**Figure 4:**
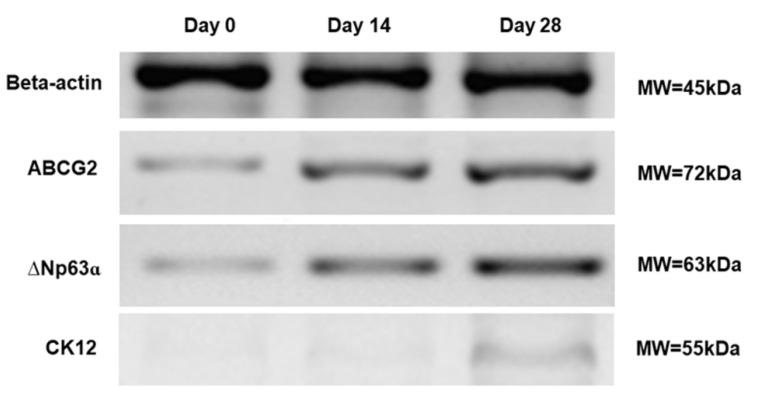
Western blot analysis during the differentiation of human iPS cells into limbal progenitor cells with the optimal BMP4 treatment. The expression levels of ABCG2 and ΔNp63α (limbal progenitor cell markers) and CK12 (corne-al epithelial cell marker) increased over time. β-actin was used as a reference protein. Representative figures from three independent experiments are shown.

Western blotting was conducted to characterize and confirm the expression profiles of human limbal progenitor and epithelial cell markers on differentiated human iPS cells. Cells cultured in PI medium with the optimal BMP4 treatment (10 ng/mL at first 3 days) exhibited elevated expression of ABCG2 and ∆Np63, limbal progenitor markers, and CK12, a corneal epithelial marker. We suggested human iPS cells could be differentiated into mixed/heterogeneous populations, including corneal epithelial and limbal progenitor cells, although there has been more population of limbal progenitors via optimal BMP4 treatment than other doses and durations of BMP4. In contrast, the expression of Nanog, a human iPS cell marker, was lower at 4 weeks (Figure 4).

### 3.5. Optimal BMP4 Treatment Favors Limbal Progenitor Cell Differentiation of Human iPS Cells

**Figure 5:**
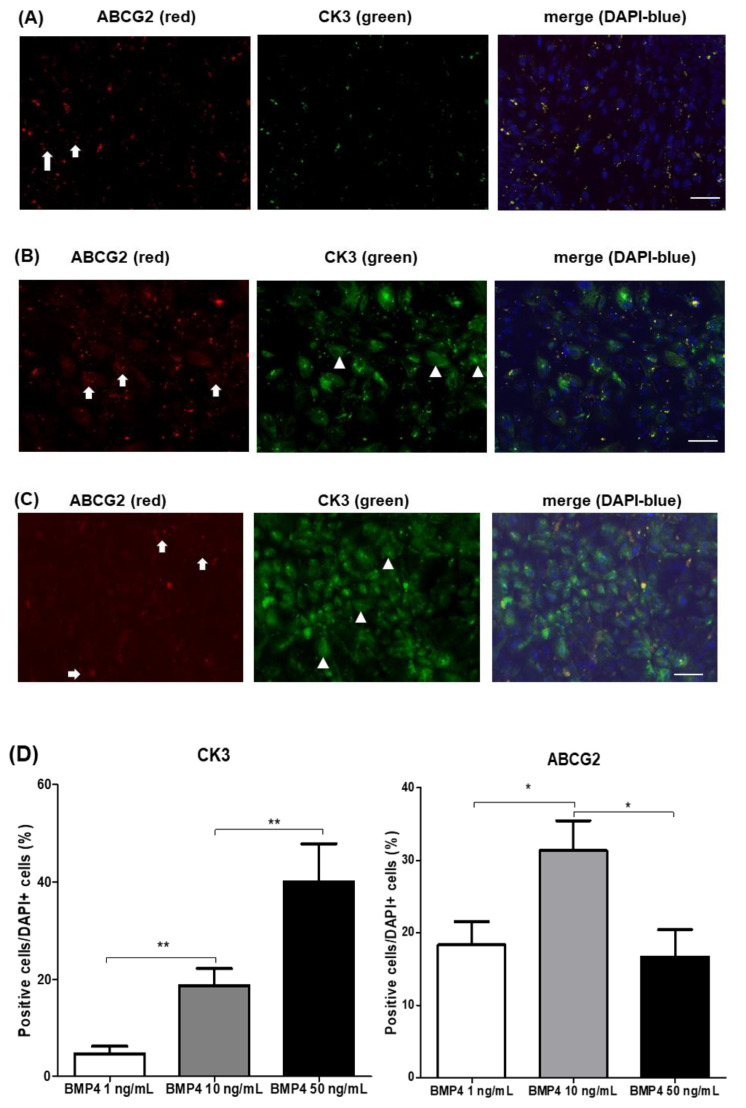
Immunohistochemical analysis of human iPS cells, differentiated with indicated concentrations of BMP4. Limbal-specific medium with 10 ng/mL BMP4 induced more limbal progenitor cells (ABCG2+, arrow) and less corneal epithelial cells (CK3+, arrowhead) on the differentiation from human iPS cells (**B**) than 1 ng/mL (**A**) or 50 ng/mL (**C**) BMP4. Images represent 3 or 4 independent experiments. Scale bar = 10 μm. (**D**) Graphs represent ≥ 3 independent experiments, and data are presented as means ± standard deviation (* *p* < 0.05; ** *p* < 0.01).

BMP4-induced differentiation of human iPS cells increased the expression of CK3, a corneal epithelial marker, in a dose-dependent manner. Notably, 10 ng/mL BMP4 increased ABCG2 and less CK3 expression significantly relative to 50 ng/mL BMP4 (*p* = 0.0125, and *p* = 0.0213, respectively; Figure 5). 10 ng/mL BMP4 treatment had higher differentiation efficiency with 31.3 ± 2.4% ABCG2-positive cells, rather than those of 1 ng/mL BMP4 (18.3 ± 1.8%) or 50 ng/mL BMP4 (16.7 ± 2.2%) group, after 1 month of culture at a density of 1 × 10^4^ iPS cells per well.

## 4. Discussion

This study established an effective strategy for differentiating human iPS cells into corneal limbal progenitor cells in vitro using limbal-specific media and an optimized BMP4 treatment protocol. BMP4 has been previously shown to differentiate iPS cells into corneal epithelial cells. Still, an optimal protocol for using BMP4 to differentiate human iPS cells into corneal limbal progenitor cells had not been reported [11,13].

LSCD is a loss of stem cells or progenitor cells from the basal cell layer of the limbus. Such cells are critical to the continuous regeneration of the corneal epithelium and providing a barrier that prevents conjunctival fibrovascular growth over the corneal surface [1,2,3]. LSCD progresses from corneal epithelial defects, conjunctival ingrowth, vascularization, and corneal opacity to vision loss. Conventional surgical interventions for LSCD include limbal transplantation (autograft or allograft from a living individual) and keratolimbal allograft from a cadaveric donor eye [3,4,20]. However, these surgical treatments are time-consuming, difficult to perform, and often require the patient to take immunosuppressive agents, such as oral steroids, cyclosporine, tacrolimus, rapamycin, and mycophenolate mofetil, for up to 2 years to control graft rejection [20,21,22]. Furthermore, most of these surgeries have failed to regenerate the ocular surface and necessitated repeated limbal transplantations [15,20,21,22,23]. Reinhard et al. demonstrated that human leukocyte antigen (HLA) matching is vital for limbal transplantation survival. Survival rates of 65% of grafts were reported with 0 or 1 mismatch, 41% with 2 to 6 mismatches, and 14% with unmatched grafts [24].

Transplantation of in vitro-cultivated limbal cells has been developed to treat LSCD. Pelligrini et al. showed that transplantation of ex vivo-expanded autologous limbal cells obtained from the healthy eye significantly improved the patient’s vision [25]. Several techniques involving ex vivo-cultivated autologous stem cell transplantation have successfully treated LSCD without the need for immunosuppressive treatment [3,26,27]. However, the availability of such limbal tissue is significantly limited because of the need to use autologous cells, high cost, the time to expand the cells ex vivo, and the potential for contamination by mouse feeder cells [3,27,28]. Human iPS cells are a potential source of limbal progenitor cells to overcome these limitations to reconstructing damaged ocular surfaces. Human iPS cells have demonstrated a remarkable capacity to proliferate and differentiate into multiple cell types [29]. Numerous studies have reported that iPS and embryonic stem cells can be differentiated into neurons, cardiac myocytes, hepatocytes, retinal cells, and corneal epithelial cells [6,7,29,30,31]. Hayashi et al. reported the production of corneal epithelial cells from human iPS cells derived from human dermal fibroblasts by the stromal cell-derived inducing activity (SDIA) differentiation method [32]. Mikhailova et al. demonstrated that human iPS cells could be terminally differentiated into mature corneal epithelial-like cells by inhibiting the TGF-β and the canonical Wnt pathways [31]. However, few established strategies to differentiate human iPS cells into corneal limbal progenitor cells have been described. Sareen et al. demonstrated that limbal cell-derived iPSCs differentiated into limbal-like cells more readily than fibroblast-derived iPS cells after culturing on a denuded human amniotic membrane [4].

Culturing limbal cells typically requires a limbal cell-specific medium. Varghese et al. reported an efficient limbal culture system using a 1:1 combination of Iscove’s modified Dulbecco’s medium and Panserin 801 (represented as PI medium) [15,16]. Iscove′s modified Dulbecco’s medium is typically used to induce rapid cell proliferation. Panserin 801, along with several supplements provided by the manufacturer, is a keratinocyte-specific medium [15,33]. Fetal bovine serum is added to stimulate the stem cells during clonal proliferation [15]. This culture system helped produce human corneal limbal cells in our previous study [16]. Therefore, we applied this limbal culture medium to differentiate human iPS cells into limbal progenitor cells. We are planning ways to eliminate animal-derived materials from the culture system to prevent xeno-contamination during clinical use. In addition, colony forming efficiency and quantification of the high-expressing ∆Np63α cells also would be supportive for limbal progenitor cells after BMP4 treatment in future experiment.

BMP4 promotes surface ectodermal differentiation. BMP4 signaling also participates in corneal epithelial cell proliferation and differentiation [11,12,13,34]. BMP4 has been reported to improve the differentiation of iPS cells to surface cells of ectodermal origin together with Wnt inhibition, such as dental or corneal epithelium [11,13]. Thus, the effect of Wnt modulation will provide the function of Wnt on the human iPS differentiation to corneal limbal and epithelial cells in our next study. However, the effects of varying BMP4 dose and duration on the differentiation of human iPS cells into limbal progenitor cells have remained unreported. In this study, human iPS cells were exposed to varying quantities (1, 10, or 50 ng/mL) and durations (1, 3, or 10 days) of BMP4 in a limbal-specific medium. The highest dose (50 ng/mL) induced higher expression of corneal epithelial markers (CK3 and CK12) and lower expression of limbal progenitor cells markers (ABCG2 and ∆Np63α) at 1 month than 10 ng/mL [19,35,36,37]. Human iPS cells could be differentiated into heterogeneous populations, including corneal epithelial and limbal progenitor cells over time, according to our Western blot and immunohistochemical analysis, although there has been more population of limbal progenitors via optimal BMP4 treatment than other doses and durations of BMP4.

Moreover, 10 days of BMP4 treatment elicited higher expression of corneal epithelial markers and lower expression of limbal progenitor cells markers than three days. Using these findings, we optimized the dose and duration of BMP4 to favor the differentiation of human iPS cells into limbal progenitor cells over corneal epithelial cells. Furthermore, the optimal BMP4 treatment maintained the limbal progenitor cell phenotype during the differentiation process by inhibiting differentiation into corneal epithelial cells. Hayashi et al. demonstrated that BMP4 suppresses iPS cells from differentiating into corneal epithelial, retinal pigment epithelial, and lens epithelial cells, related to due to BMP4 inhibiting early Pax6 upregulation [32]. Therefore, the changes and functions of Pax6 expression according to the different doses of BMP4 will be analyzed in our further experiments. Kobayashi Y et al. reported that the first four days of BMP4 treatment are critical for determining the fate of iPS cell differentiation into ocular surface ectodermal cells [11]. Treatments lasting longer than four days did not significantly affect the pattern of differentiation. The concentration and duration of BMP4 treatment are important factors for differentiating human iPS cells into limbal progenitor and corneal epithelial cells.

## 5. Conclusions

This study provides evidence that human dermal fibroblast-derived iPS cells can differentiate to putative limbal progenitor cells in limbal specific media and an optimal BMP4 treatment, based upon data acquired from varying the dose and duration of BMP4 exposure. Moreover, our study suggests that banks of HLA-typed limbal stem cells differentiated from human iPS cells could be established for therapeutic application to LSCD in the future.

## Figures and Tables

**Table 1:** List of antibodies and dilutions for Western blot assay.

Primary/Secondary Antibody	Host	Dilution	Manufacturer
mouse anti-ABCG2 (BXP21)	Mouse	1:1000	Santa cruz (sc-58222)
rabbit anti-ΔNp63 (4A4)	Rabbit	1:1000	Santa cruz (sc-8431)
goat-anti-cytokeratin 12	Goat	1:1000	Santa cruz (sc-17101)
rabbit anti-beta-actin	Rabbit	1:1000	Abcam (ab8227)
anti-mouse IgG-HRP	Donkey	1:2000	Santa cruz (sc-2314)
anti-rabbit IgG-HRP	Goat	1:2000	Santa cruz (sc-2004)
anti-goat IgG-HRP	Donkey	1:2000	Santa cruz (sc-2020)

## Data Availability

The data used in this study are available from the corresponding author upon reasonable request.

## References

[1] Oie Y., Nishid K. (2014). Translational research on ocular surface reconstruction using oral mucosal epithelial cell sheets. Cornea.

[2] Ramos T., Scott D., Ahmad S. (2015). An Update on Ocular Surface Epithelial Stem Cells: Cornea and Conjunctiva. Stem Cells Int..

[3] Nakamura T., Kinoshita S. (2011). New hopes and strategies for the treatment of severe ocular surface disease. Curr. Opin. Ophthalmol..

[4] Sareen D., Saghizadeh M., Ornelas L., Winkler M.A., Narwani K., Sahabian A., Funari V.A., Tang J., Spurka L., Punj V. (2014). Differentiation of human limbal-derived induced pluripotent stem cells into limbal-like epithelium. Stem Cells Transl. Med..

[5] Ahmad S. (2012). Concise review: Limbal stem cell deficiency, dysfunction, and distress. Stem Cells Transl. Med..

[6] Inoue H., Nagata N., Kurokawa H., Yamanaka S. (2014). iPS cells: A game changer for future medicine. EMBO J..

[7] Wetsel R.A., Wang D., Calame D.G. (2011). Therapeutic potential of lung epithelial progenitor cells derived from embryonic and induced pluripotent stem cells. Annu. Rev. Med..

[8] Juuti-Uusitalo K., Delporte C., Grégoire F., Perret J., Huhtala H., Savolainen V., Nymark S., Hyttinen J., Uusitalo H., Willermain F. (2013). Aquaporin expression and function in human pluripotent stem cell-derived retinal pigmented epithelial cells. Invest. Ophthalmol. Vis. Sci..

[9] Rubinstein T.J., Weber A.C., Traboulsi E.I. (2016). Molecular biology and genetics of embryonic eyelid development. Ophthalmic Genet..

[10] Ito Y.A., Walter M.A. (2014). Genomics and anterior segment dysgenesis: A review. Clin. Exp. Ophthalmol..

[11] Kobayashi Y., Hayashi R., Shibata S., Quantock A.J., Nishida K. (2020). Ocular surface ectoderm instigated by WNT inhibition and BMP4. Stem Cell Res..

[12] Zhang Y., Yeh L.K., Zhang S., Call M., Yuan Y., Yasunaga M., Kao W.W., Liu C.Y. (2015). Wnt/β-catenin signaling modulates corneal epithelium stratification via inhibition of Bmp4 during mouse development. Development.

[13] Hayashi R., Ishikawa Y., Sasamoto Y., Katori R., Nomura N., Ichikawa T., Araki S., Soma T., Kawasaki S., Sekiguchi K. (2016). Co-ordinated ocular development from human iPS cells and recovery of corneal function. Nature.

[14] Soga M., Ishitsuka Y., Hamasaki M., Yoneda K., Furuya H., Matsuo M., Ihn H., Fusaki N., Nakamura K., Nakagata N. (2015). HPGCD outperforms HPBCD as a potential treatment for Niemann-Pick disease type C during disease modeling with iPS cells. Stem Cells.

[15] Varghese V.M., Prasad T., Kumary T.V. (2010). Optimization of culture conditions for an efficient xeno-feeder free limbal cell culture system towards ocular surface regeneration. Microsc. Res. Tech..

[16] Na K.S., Mok J.W., Joo C.K. (2015). Ex vivo human corneal epithelial cell expansion from a xeno-feeder-free system. Ophthalmic Res..

[17] Zhang M., Ngo J., Pirozzi F., Sun Y.P., Wynshaw-Boris A. (2018). Highly efficient methods to obtain homogeneous dorsal neural progenitor cells from human and mouse embryonic stem cells and induced pluripotent stem cells. Stem Cell Res Ther..

[18] Wakui T., Matsumoto T., Matsubara K., Kawasaki T., Yamaguchi H., Akutsu H. (2017). Method for evaluation of human induced pluripotent stem cell quality using image analysis based on the biological morphology of cells. J. Med. Imaging.

[19] Notara M., Daniels J.T. (2010). Characterization and functional features of a spontaneously immortalised human corneal epithelial cell line with progenitor-like characteristics. Brain Res. Bull..

[20] Dua H.S., Miri A., Said D.G. (2010). Contemporary limbal stem cell transplantation—A review. Clin. Exp. Ophthalmol..

[21] Tsubota K., Satake Y., Kaido M., Shinozaki N., Shimmura S., Bissen-Miyajima H., Shimazaki J. (1999). Treatment of severe ocular-surface disorders with corneal epithelial stem cell transplantation. N. Engl. J. Med..

[22] Joseph A., Raj D., Shanmuganathan V., Powell R.J., Dua H.S. (2007). Tacrolimus immunosuppression in high-risk corneal grafts. Br. J. Ophthalmol..

[23] Ang A.Y., Chan C.C., Biber J.M., Holland E.J. (2013). Ocular surface stem cell transplantation rejection: Incidence, characteristics, and outcomes. Cornea.

[24] Reinhard T., Spelsberg H., Henke L., Kontopoulos T., Enczmann J., Wernet P., Berschick P., Sundmacher R., Böhringer D. (2004). Long-term results of allogeneic penetrating limbo-keratoplasty in total limbal stem cell deficiency. Ophthalmology.

[25] Pellegrini G., Traverso C.E., Franzi A.T., Zingirian M., Cancedda R., De Luca M. (1997). Long-term restoration of damaged corneal surfaces with autologous cultivated corneal epithelium. Lancet.

[26] Nakamura T., Sotozono C., Bentley A.J., Mano S., Inatomi T., Koizumi N., Fullwood N.J., Kinoshita S. (2010). Long-term phenotypic study after allogeneic cultivated corneal limbal epithelial transplantation for severe ocular surface diseases. Ophthalmology.

[27] Di Girolamo N., Bosch M., Zamora K., Coroneo M.T., Wakefield D., Watson S.L. (2009). A contact lens-based technique for expansion and transplantation of autologous epithelial progenitors for ocular surface reconstruction. Transplantation.

[28] Casaroli-Marano R.P., Nieto-Nicolau N., Martínez-Conesa E.M., Edel M., Álvarez-Palomo A.B. (2015). Potential role of induced pluripotent stem cells for cell-based therapy of the ocular surface. J. Clin. Med..

[29] Yamanaka S. (2012). Induced pluripotent stem cells: Past, present, and future. Cell Stem Cell.

[30] Mekala S.R., Vauhini V., Nagarajan U., Maddileti S., Gaddipati S., Mariappan I. (2013). Derivation, characterization and retinal differentiation of induced pluripotent stem cells. J. Biosci..

[31] Mikhailova A., Ilmarinen T., Uusitalo H., Skottman H. (2014). Small-molecule induction promotes corneal epithelial cell differentiation from human induced pluripotent stem cells. Stem Cell Rep..

[32] Hayashi R., Ishikawa Y., Ito M., Kageyama T., Takashiba K., Fujioka T., Tsujikawa M., Miyoshi H., Yamato M., Nakamura Y. (2012). Generation of corneal epithelial cells from induced pluripotent stem cells derived from human dermal fibroblast and corneal limbal epithelium. PLoS ONE.

[33] Varani J., Mitra R.S., Gibbs D., Phan S.H., Dixit V.M., Mitra R., Wang T., Siebert K.J., Nickoloff B.J., Voorhees J.J. (1999). All-trans retinoic acid stimulates growth and extracellular matrix production in growth-inhibited cultured human skin fibroblasts. J. Investig. Dermatol..

[34] Wilson P.A., Hemmati-Brivanlou A. (1995). Induction of epidermis and inhibition of neural fate by Bmp-4. Nature.

[35] Sakurai M., Hayashi R., Kageyama T., Yamato M., Nishida K. (2011). Induction of putative stratified epithelial progenitor cells in vitro from mouse-induced pluripotent stem cells. J. Artif. Organs..

[36] Chee K.Y., Kicic A., Wiffen S.J. (2006). Limbal stem cells: The search for a marker. Clin. Exp. Ophthalmol..

[37] Epstein S.P., Wolosin J.M., Asbell P.A. (2005). P63 expression levels in side population and low light scattering ocular surface epithelial cells. Trans. Am. Ophthalmol. Soc..

